# Supplementary material for: Grik2b and Grik2c kainate receptors regulate oviposition in Bactrocera dorsalis
Source: PLoS Biol. 2026 Feb 2;24(2):e3003609. doi: 10.1371/journal.pbio.3003609 (PMC12875582; doi:10.1371/journal.pbio.3003609)
Supplement: S1 Fig — (A) PCA reveals variations in gene expression across different developmental stages of female ovipositors. (B) Ovipositor differentially expressed genes between 12-day old females and newly emerged females (0-day old). (C) Ovipositor differentially expressed genes between 12-day old females and 3-day old females. (D) Ovipositor differentially expressed genes between 12-day old females and 6-day old females. (E) Ovipositor differentially expressed genes between 12-day old females and 9-day old females. (DOCX) [file pbio.3003609.s001.docx]

**
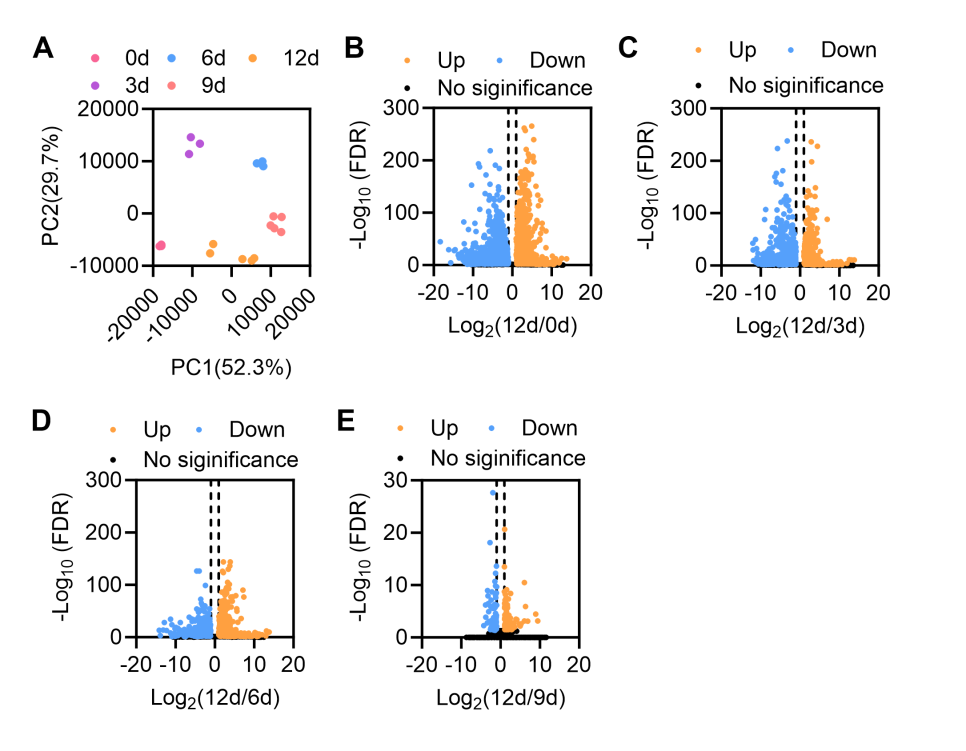
**

**S1 Fig. Differential gene expression in the ovipositor of gravid females compared to that of younger females.**

**(A)** PCA reveals variations in gene expression across different developmental stages of female ovipositors.

**(B)** Ovipositor differentially expressed genes between 12-day old females and newly emerged females (0-day old).

**(C)** Ovipositor differentially expressed genes between 12-day old females and 3-day old females. **(D)** Ovipositor differentially expressed genes between 12-day old females and 6-day old females. **(E)** Ovipositor differentially expressed genes between 12-day old females and 9-day old females.
