# Supplementary figures and images for: Grik2b and Grik2c kainate receptors regulate oviposition in Bactrocera dorsalis
Source: PLoS Biol. 2026 Feb 2;24(2):e3003609. doi: 10.1371/journal.pbio.3003609 (PMC12875582; doi:10.1371/journal.pbio.3003609)

**
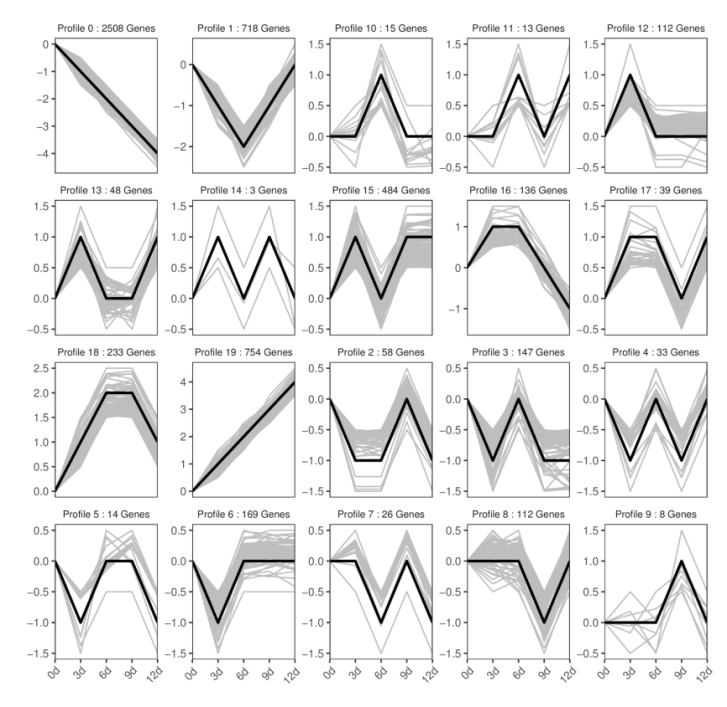
**

**S2 Fig. Expression trend of ovipositor genes at different development stages.**

Supplement: S2 Fig — (DOCX) [file pbio.3003609.s002.docx]

**
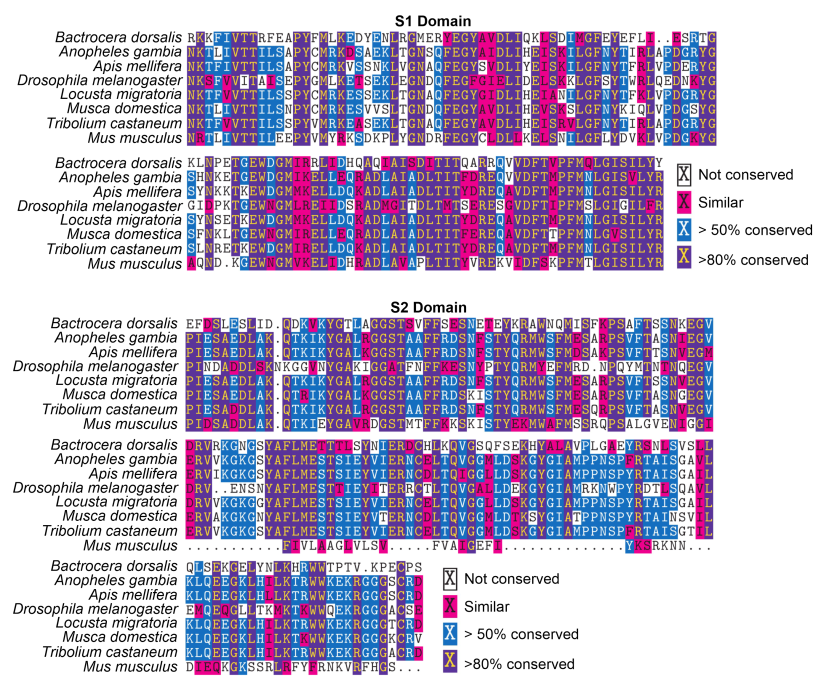
**

**S3 Fig. Similarity of the ligand-binding domains of Grik2b to those found in other insects.**

Supplement: S3 Fig — (DOCX) [file pbio.3003609.s003.docx]

**
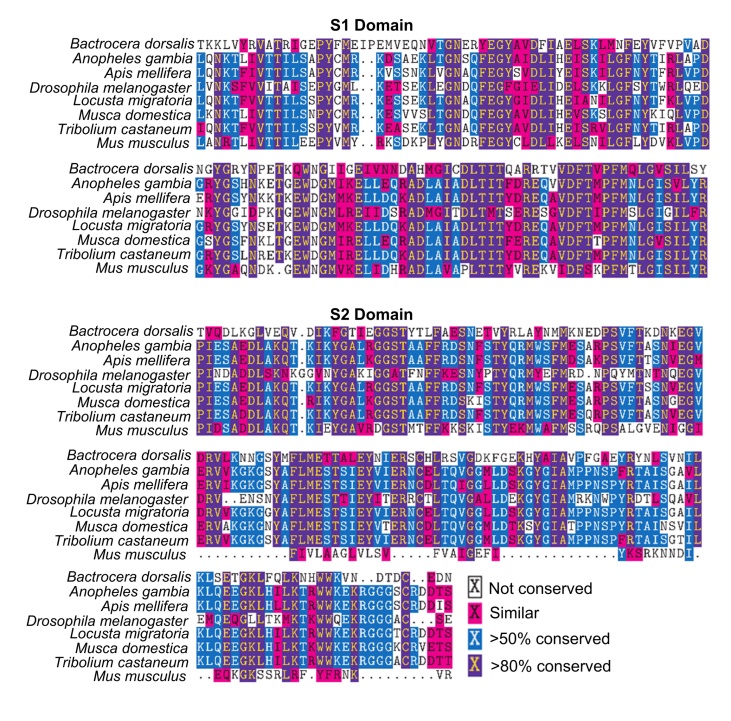
**

**S4 Fig. Similarity of the ligand-binding domains of Grik2c to those found in other insects.**

Supplement: S4 Fig — (DOCX) [file pbio.3003609.s004.docx]

**
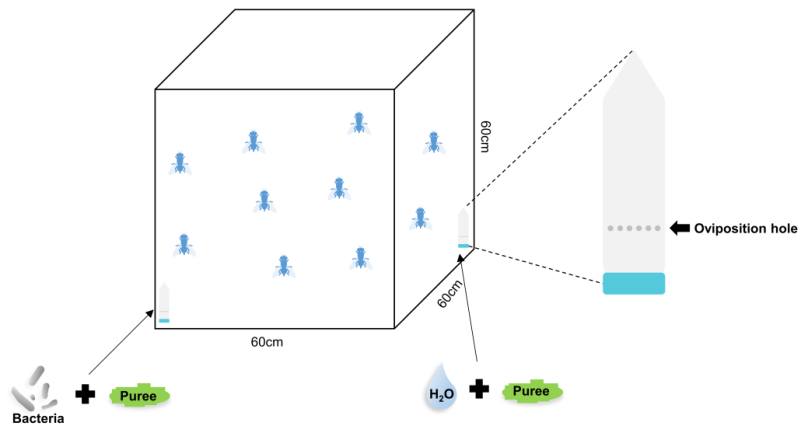
**

**S6 Fig. A device used for testing oviposition preference.**

Supplement: S6 Fig — (DOCX) [file pbio.3003609.s006.docx]

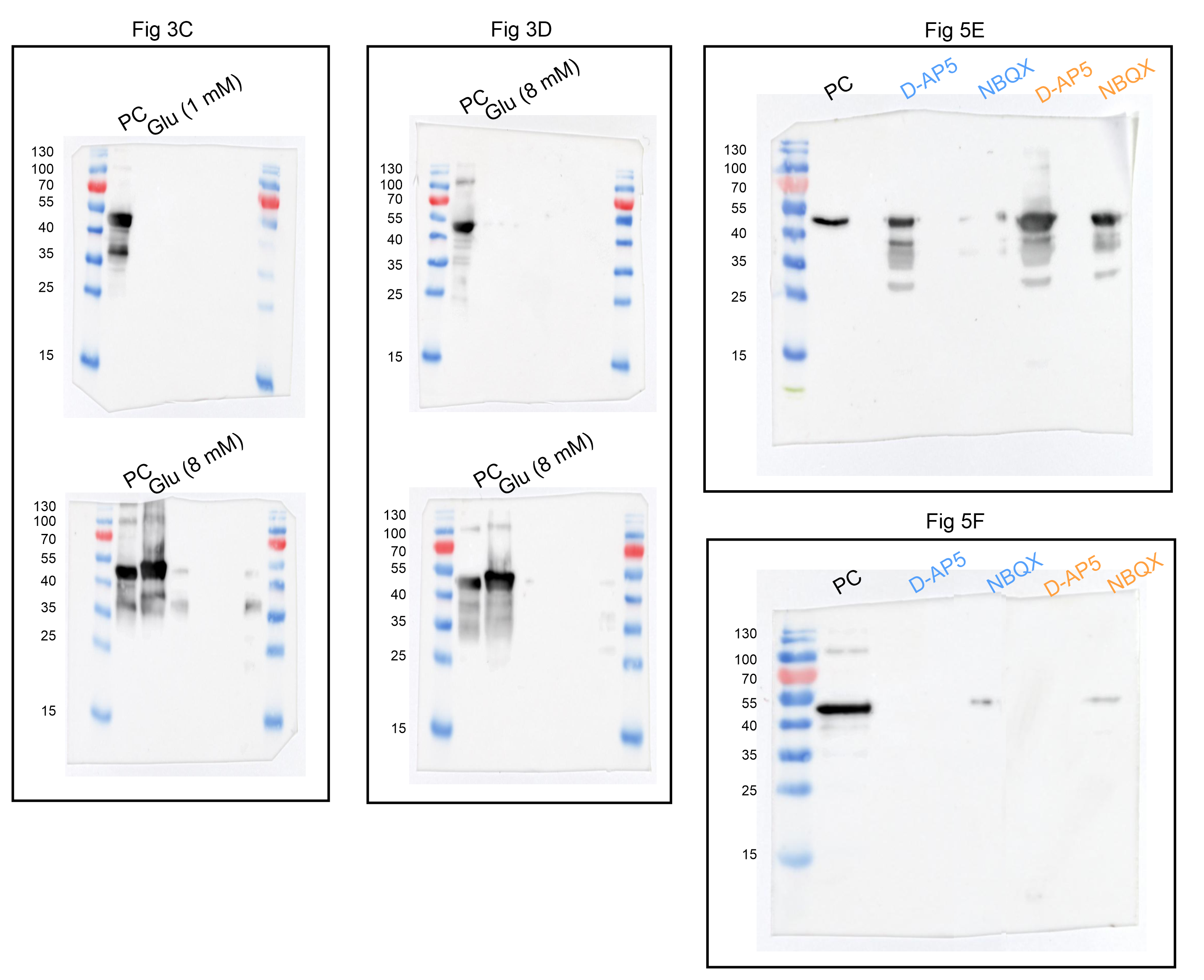

Supplement: S1 Raw Images — (TIF) [file pbio.3003609.s028.tif]
