# Supplementary material for: Grik2b and Grik2c kainate receptors regulate oviposition in Bactrocera dorsalis
Source: PLoS Biol. 2026 Feb 2;24(2):e3003609. doi: 10.1371/journal.pbio.3003609 (PMC12875582; doi:10.1371/journal.pbio.3003609)
Supplement: S5 Fig — (A) Hematoxylin and Eosin (HE) staining showed the muscle cells were enveloped by neurons in ovipositor. (B) Muscle and neuron fibers labeled by Phalloidin (red) and Horseradish Peroxidase (HRP) (green), respectively, in the ovipositor. (C) Muscle and neuron cells labeled by Phalloidin (red) and HRP (green), respectively, in the ovipositor. The nucleus is stained with DAPI (blue). (D) NMJ observed by transmission electron microscopy in the ovipositor. (E) No Grik2b and Grik2c signal (red) identified in NMJs of the ovipositor by FISH with sense probes. (F) Weak Grik2b and Grik2c signal identified in NMJs of the ovipositor by FISH after RNase treatment. (DOCX) [file pbio.3003609.s005.docx]

**
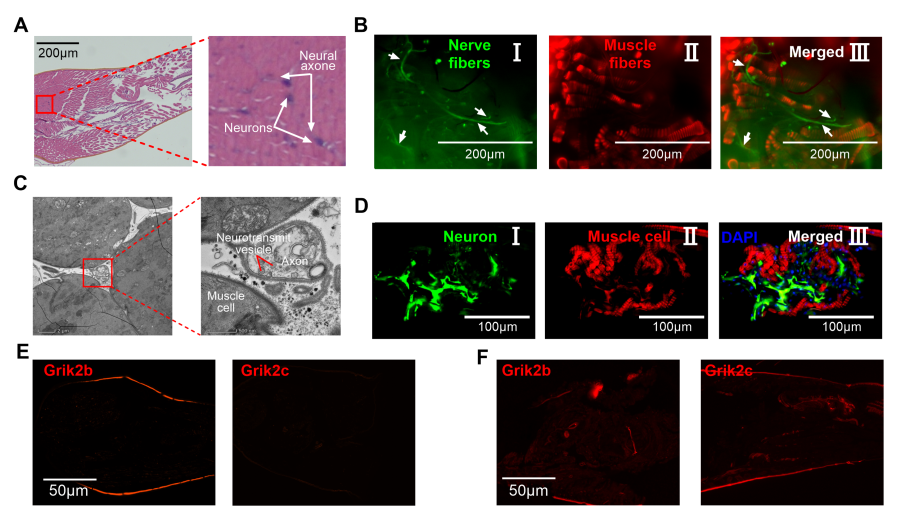
**

**S5 Fig. Microscopic observation of the internal structure of the ovipositor.**

**(A)** Haematoxylin and Eosin (HE) staining showed the muscle cells were enveloped by neurons in ovipositor.

**(B)** Muscle and neuron fibers labeled by Phalloidin (red) and Horseradish Peroxidase (HRP) (green), respectively, in the ovipositor.

**(C)** Muscle and neuron cells labeled by Phalloidin (red) and HRP (green), respectively, in the ovipositor. The nucleus is stained with DAPI (blue).

**(D)** NMJ observed by transmission electron microscopy in the ovipositor.

**(E)** No Grik2b and Grik2c signal (red) identified in NMJs of the ovipositor by FISH with sense probes.

**(F)** Weak Grik2b and Grik2c signal identified in NMJs of the ovipositor by FISH after RNase treatment.
