# Supplementary material for: Grik2b and Grik2c kainate receptors regulate oviposition in Bactrocera dorsalis
Source: PLoS Biol. 2026 Feb 2;24(2):e3003609. doi: 10.1371/journal.pbio.3003609 (PMC12875582; doi:10.1371/journal.pbio.3003609)
Supplement: S7 Fig — (A) Expression of Grik2b in ovipositor 72 h after Grik2b dsRNA injection (n = 5, P = 0.0237, Independent sample student t test). (B) Expression of Grik2c in ovipositor 72 h after Grik2b dsRNA injection (n = 5, P = 0.8521, Independent sample student t test). (C) Expression of Grik2c in ovipositor 72 h after Grik2c dsRNA injection (n = 5, P = 0.0211, Independent sample student t test). (D) Expression of Grik2b in ovipositor 72 h after Grik2c dsRNA injection (n = 5, P = 0.9433, Independent sample student t test). (E) Eggs in ovary of female after Grik2b/c being knockdown (n = 13, F(2,36) = 0.6926, P = 0.5068, Ordinary one-way ANOVA). (F) Expression of Grik2b in ovipositor 72 h after additional multiple non-overlapping RNAi constructs injection (n = 5, P = 0.0002, Independent sample student t test). (G) Expression of Grik2c in ovipositor 72 h after additional multiple non-overlapping RNAi constructs injection (n = 5, P = 0.0002, Independent sample student t test). (H) Total eggs laid by gravid females injected with additional multiple non-overlapping RNAi constructs (n = 15, F(2,84) = 0.7331, P < 0.0001, Two-way ANOVA). (I) Oviposition preference of females injected with additional multiple non-overlapping RNAi constructs (dsGFP: n = 16, P < 0.0001; dsGrik2b: n = 16, P = 0.2401; dsGrik2c: i = 16, P = 0.969; Paired sample student t test). (J) Expression of Grik2a in ovipositor 72 h after Grik2a dsRNA injection (n = 4, P = 0.0009, Independent sample student t test). (K) Total eggs laid by gravid females injected with Grik2a RNAi constructs (n = 15, F(1,56) = 3.373, P = 0.0583, Two-way ANOVA). (L) Oviposition preference of females injected with with Grik2a RNAi constructs. (dsGFP: n = 16, P < 0.0001; dsGrik2a: n = 16, P = 0.0079; Paired sample student t test). The data underlying this figure can be found in S6 Data. (DOCX) [file pbio.3003609.s007.docx]

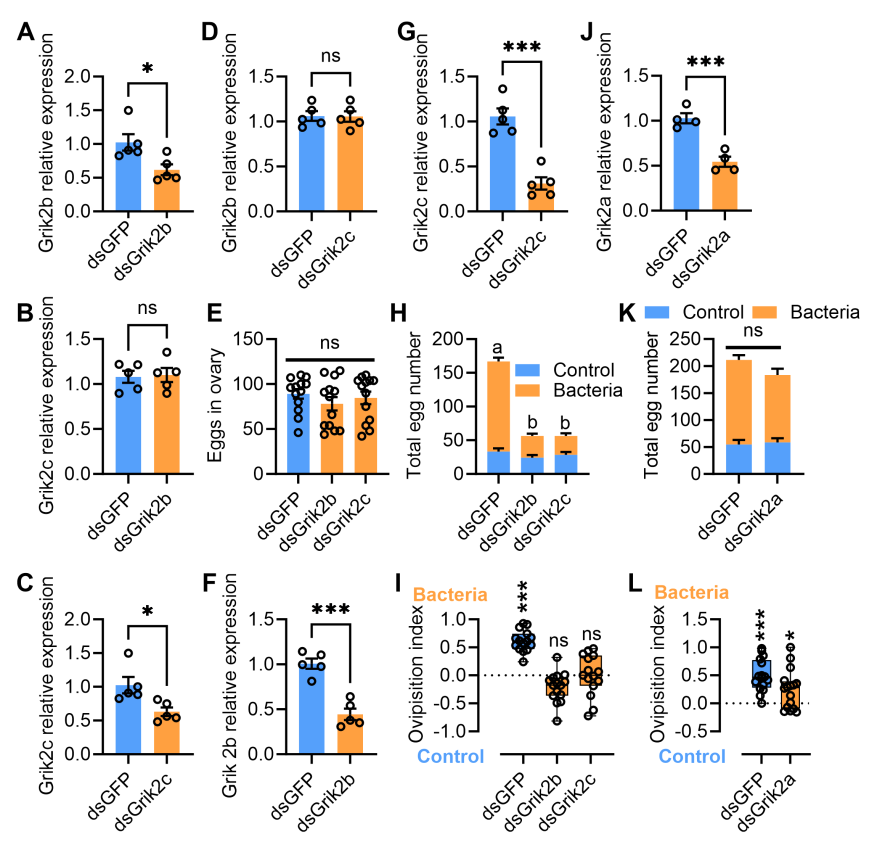


**S7 Fig. Influence of Grik2a, Grik2b or Grik2c knockdown on oviposition.**

**(A)** Expression of Grik2b in ovipositor 72h after Grik2b dsRNA injection (n = 5, *P* = 0.0237, Independent sample student’s *t* test).

**(B)** Expression of Grik2c in ovipositor 72h after Grik2b dsRNA injection (n = 5, *P* = 0.8521, Independent sample student’s *t* test).

**(C)** Expression of Grik2c in ovipositor 72h after Grik2c dsRNA injection (n = 5, *P* = 0.0211, Independent sample student’s *t* test).

**(D)** Expression of Grik2b in ovipositor 72h after Grik2c dsRNA injection (n = 5, *P* = 0.9433, Independent sample student’s *t* test).

**(E)** Eggs in ovary of female after Grik2b/c being knockdown (n = 13, *F*_(2,36)_ = 0.6926, *P* = 0.5068, Ordinary one-way ANOVA).

**(F)** Expression of Grik2b in ovipositor 72h after additional multiple non overlapping RNAi constructs injection (n = 5, *P* = 0.0002, Independent sample student’s *t* test).

**(G)** Expression of Grik2c in ovipositor 72h after additional multiple non overlapping RNAi constructs injection (n = 5, *P* = 0.0002, Independent sample student’s *t* test).

**(H)** Total eggs laid by gravid females injected with additional multiple non overlapping RNAi constructs (n = 15, *F*_(2,84)_ = 0.7331, *P* < 0.0001, Two-way ANOVA).

**(I)** Oviposition preference of females injected with additional multiple non overlapping RNAi constructs (dsGFP: n = 16, *P* < 0.0001; dsGrik2b: n = 16, *P* = 0.2401; dsGrik2c: n = 16, *P* = 0.969; Paired sample student’s *t* test).

**(J)** Expression of Grik2a in ovipositor 72h after Grik2a dsRNA injection (n = 4, *P* = 0.0009, Independent sample student’s *t* test).

**(K)** Total eggs laid by gravid females injected with Grik2a RNAi constructs (n = 15, *F*_(1,56)_ = 3.373, *P* = 0.0583, Two-way ANOVA).

**(L)** Oviposition preference of females injected with with Grik2a RNAi constructs. (dsGFP: n = 16, *P* < 0.0001; dsGrik2a: n = 16, *P* = 0.0079; Paired sample student’s *t* test).

The data underlying this figure can be found in S6 Data.
