# Supplementary material for: Grik2b and Grik2c kainate receptors regulate oviposition in Bactrocera dorsalis
Source: PLoS Biol. 2026 Feb 2;24(2):e3003609. doi: 10.1371/journal.pbio.3003609 (PMC12875582; doi:10.1371/journal.pbio.3003609)
Supplement: S8 Fig — (A) Example of female movement trajectories and heat maps. (B) Move distance comparison between KAR, GluDH, GluSN knocked down females and the control (n = 12, F(4,55) = 0.3695, P = 0.8294, One-way ANOVA). (C) Move speed comparison between KAR, GluDH, GluSN knocked down females and the control (n = 12, F(4,55) = 0.3847, P = 0.8187, One-way ANOVA). (D) Manic time comparison between KAR, GluDH, GluSN knocked down females and the control (n = 12, F(4,55) = 0.4673, P = 0.7595, One-way ANOVA). (E) Manic frequency comparison between KAR, GluDH, GluSN knocked down females and the control (n = 12, F(4,55) = 0.5589, P = 0.6934, One-way ANOVA). (F) Active time comparison between KAR, GluDH, GluSN knocked down females and the control (n = 12, F(4,55) = 0.2834, P = 0.8875, One-way ANOVA). (G) Active frequence comparison between KAR, GluDH, GluSN knocked down females and the control (n = 12, F(4,55) = 0.2707, P = 0.8956, One-way ANOVA). (H) Still time comparison between KAR, GluDH, GluSN knocked down females and the control (n = 12, F(4,55) = 0.713, P = 0.5866, One-way ANOVA). (I) Still frequence comparison between KAR, GluDH, GluSN knocked down females and the control (n = 12, F(4,55) = 0.2853, P = 0.8863, One-way ANOVA). The data underlying this figure can be found in S7 Data. (DOCX) [file pbio.3003609.s008.docx]

**
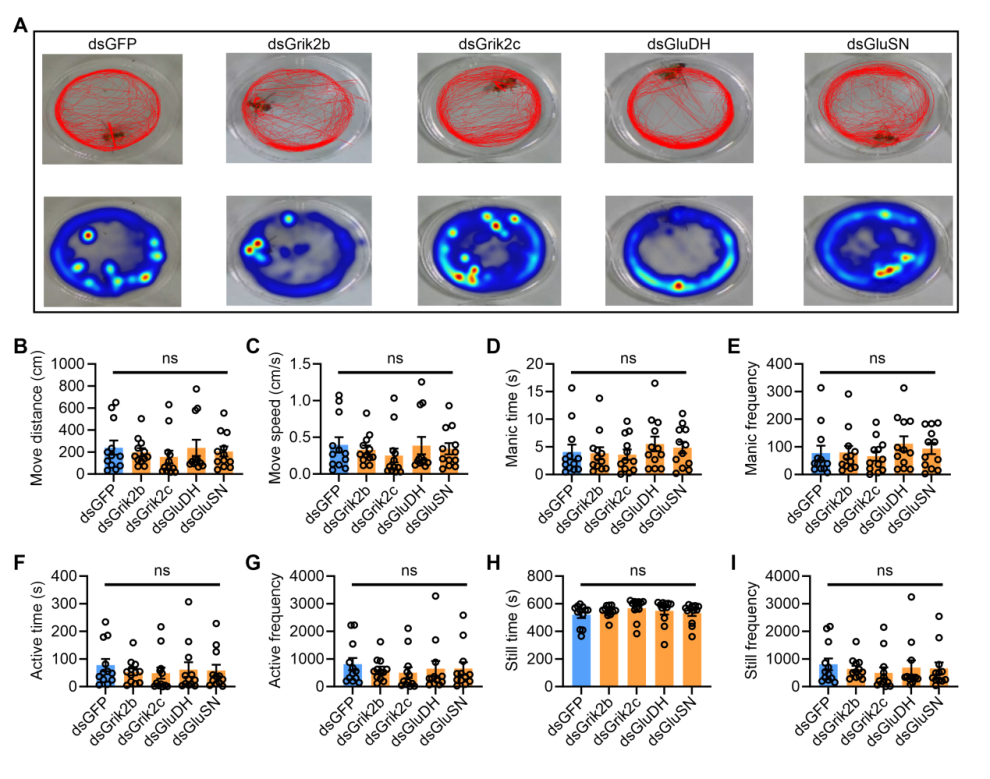
**

**S8 Fig. Motion ability of the females with the KAR and glutamate synthesis genes knocked down.**

**(A)** Example of female movement trajectories and heat maps.

**(B)** Move distance comparison between KAR, GluDH, GluSN knocked down females and the control (n = 12, *F*_(4,55)_ = 0.3695, *P* = 0.8294, One-way ANOVA).

**(C)** Move speed comparison between KAR, GluDH, GluSN knocked down females and the control (n = 12, *F*_(4,55)_ = 0.3847, *P* = 0.8187, One-way ANOVA).

**(D)** Manic time comparison between KAR, GluDH, GluSN knocked down females and the control (n = 12, *F*_(4,55)_ = 0.4673, *P* = 0.7595, One-way ANOVA).

**(E)** Manic frequency comparison between KAR, GluDH, GluSN knocked down females and the control (n = 12, *F*_(4,55)_ = 0.5589, *P* = 0.6934, One-way ANOVA).

**(F)** Active time comparison between KAR, GluDH, GluSN knocked down females and the control (n = 12, *F*_(4,55)_ = 0.2834, *P* = 0.8875, One-way ANOVA).

**(G)** Active frequence comparison between KAR, GluDH, GluSN knocked down females and the control (n = 12, *F*_(4,55)_ = 0.2707, *P* = 0.8956, One-way ANOVA).

**(H)** Still time comparison between KAR, GluDH, GluSN knocked down females and the control (n = 12, *F*_(4,55)_ = 0.713, *P* = 0.5866, One-way ANOVA).

**(I)** Still frequence comparison between KAR, GluDH, GluSN knocked down females and the control (n = 12, *F*_(4,55)_ = 0.2853, *P* = 0.8863, One-way ANOVA).

The data underlying this figure can be found in S7 Data.
