# Supplementary material for: Grik2b and Grik2c kainate receptors regulate oviposition in Bactrocera dorsalis
Source: PLoS Biol. 2026 Feb 2;24(2):e3003609. doi: 10.1371/journal.pbio.3003609 (PMC12875582; doi:10.1371/journal.pbio.3003609)
Supplement: S9 Fig — (A) Effect of knocking down muscle development and contraction genes on egg-laying (n = 10, F(4,45) = 16.5, P < 0.001, One-way ANOVA). (B–E) Effect of Grik2b/c knockdown on the expression of muscle development and contraction genes (B: n = 3, F(2,6) = 0.1413, P = 0.871; C: n = 3, F(2,3) = 0.7379, P = 0.517; D: n = 3, F(2,6) = 0.0618, P = 0.9406; E: n = 3, F(2,6) = 0.0156, P = 0.9845; One-way ANOVA). The data underlying this figure can be found in S8 Data. (DOCX) [file pbio.3003609.s009.docx]

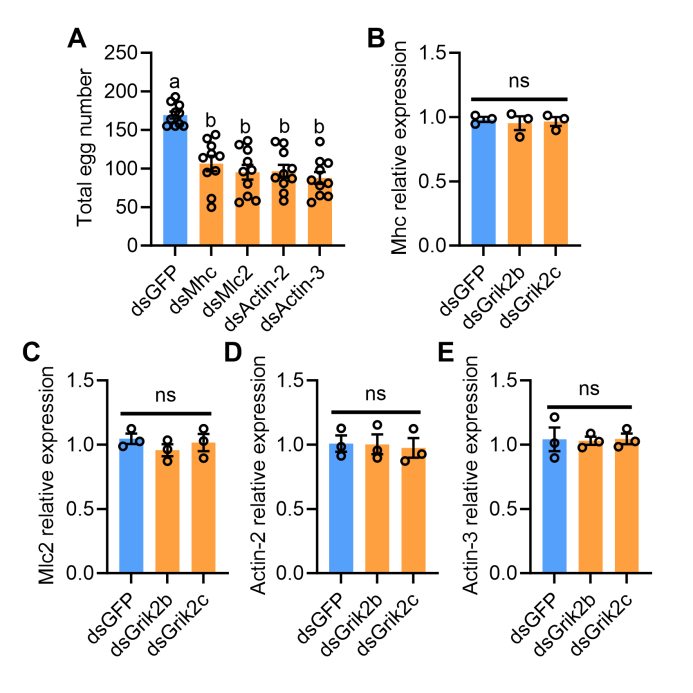


**S9 Fig. Effects of muscle development and contraction related genes on oviposition and Gria2b/c expression.**

**(A)** Effect of knocking down muscle development and contraction genes on egg-laying (n = 10, *F*_(4,45)_ = 16.5, *P* < 0.001, One-way ANOVA).

**(B-E)** Effect of Grik2b/c knockdown on the expression of muscle development and contraction genes (**B**: n = 3, *F*_(2,6)_ = 0.1413, *P* = 0.871; **C**: n = 3, *F*_(2,3)_ = 0.7379, *P* = 0.517; **D**: n = 3, *F*_(2,6)_ = 0.0618, *P* = 0.9406; **E**: n = 3, *F*_(2,6)_ = 0.0156, *P* = 0.9845; One-way ANOVA).

The data underlying this figure can be found in S8 Data.
