# Supplementary material for: Grik2b and Grik2c kainate receptors regulate oviposition in Bactrocera dorsalis
Source: PLoS Biol. 2026 Feb 2;24(2):e3003609. doi: 10.1371/journal.pbio.3003609 (PMC12875582; doi:10.1371/journal.pbio.3003609)
Supplement: S10 Fig — (A) Schematic of Grik2b LBD model. (B) The quality of the Grik2b LBD model assessed by Ramachandran Plot. (C) Schematic of Grik2c LBD model. (D) The quality of the Grik2c LBD model assessed by Ramachandran Plot. (DOCX) [file pbio.3003609.s010.docx]

**
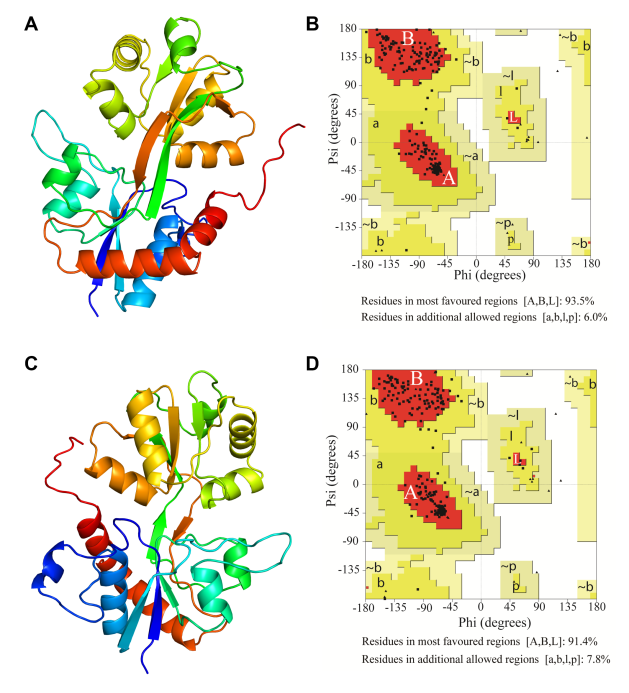
**

**S10 Fig. Construction and evaluation of LBDs of Grik2b and Grik2c model.**

**(A)** Schematic of Grik2b LBD model.

**(B)** The quality of the Grik2b LBD model assessed by Ramachandran Plot.

**(C)** Schematic of Grik2c LBD model.

**(D)** The quality of the Grik2c LBD model assessed by Ramachandran Plot.
