# Supplementary material for: Grik2b and Grik2c kainate receptors regulate oviposition in Bactrocera dorsalis
Source: PLoS Biol. 2026 Feb 2;24(2):e3003609. doi: 10.1371/journal.pbio.3003609 (PMC12875582; doi:10.1371/journal.pbio.3003609)
Supplement: S11 Fig — (A) RNAi efficiency of GluDH (n = 4, P = 0.0047, Independent sample student t test). (B) RNAi efficiency of GluSN (n = 5, P = 0.0321, Independent sample student t test). The data underlying this figure can be found in S9 Data. (DOCX) [file pbio.3003609.s011.docx]

**
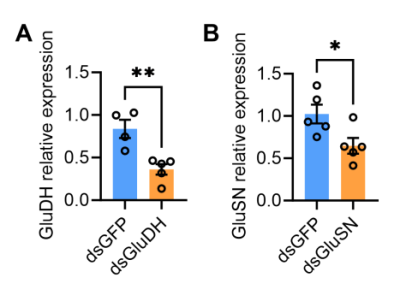
**

**S11 Fig. RNAi efficiency of the glutamate synthesis genes.**

**(A)** RNAi efficiency of GluDH (n = 4, *P* = 0.0047, Independent sample student’s *t* test).

**(B)** RNAi efficiency of GluSN (n = 5, *P* = 0.0321, Independent sample student’s *t* test).

The data underlying this figure can be found in S9 Data.
