# Supplementary material for: Grik2b and Grik2c kainate receptors regulate oviposition in Bactrocera dorsalis
Source: PLoS Biol. 2026 Feb 2;24(2):e3003609. doi: 10.1371/journal.pbio.3003609 (PMC12875582; doi:10.1371/journal.pbio.3003609)
Supplement: S12 Fig — (A) Grik2b and Grik2c expression in CNS 12 h after thorax injection of dsRNA targeting Grik2b or Grik2c (Grik2b: n = 4, P = 0.031; Grik2c: n = 4, P = 0.0042; Independent sample student t test). (B) Grik2b and Grik2c expression in ovipositor 12 h after thorax injection of dsRNA targeting Grik2b or Grik2c (Grik2b: n = 5, P = 0.8635; Grik2c: n = 5, P = 0.9123; Independent sample student t test). (C) Grik2b and Grik2c expression in ovipositor 24 h after thorax injection of dsRNA targeting Grik2b or Grik2c (Grik2b: n = 4, P < 0.001; Grik2c: n = 4, P = 0.0216; Independent sample student t test). (D) Grik2b and Grik2c expression in CNS 24 h after thorax injection of dsRNA targeting Grik2b or Grik2c (Grik2b: n = 4, P = 0.009; Grik2c: n = 4, P = 0.0144; Independent sample student t test). (E) Oviposition preference to the gut bacteria strain added fruit of females with dsRNA of Grik2b and Grik2c injected into thorax for 24 h (dsGFP: n = 10, P < 0.0001; dsGrik2b: n = 10, P = 0.9543; dsGrik2c: n = 10, P = 0.7336; Paired sample student t test). (F) Total eggs laid by females with dsRNA of Grik2b and Grik2c injected into thorax for 24 h (n = 10, F(2,54) = 5.945, P = 0.0046, Two-way ANOVA). (G) Grik2b and Grik2c expression in ovipositor 6 h after abdomen injection of dsRNA targeting Grik2b or Grik2c (Grik2b: n = 4, P < 0.001; Grik2c: n = 4, P = 0.002; Independent sample student t test). (H) Grik2b and Grik2c expression in CNS 6 h after abdomen injection of dsRNA targeting Grik2b or Grik2c (Grik2b: n = 4, P = 0.6978; Grik2c: n = 4, P = 0.2639; Independent sample student t test). (I) Grik2b and Grik2c expression in ovipositor 12 h after abdomen injection of dsRNA targeting Grik2b or Grik2c (Grik2b: n = 4, P = 0.0041; Grik2c: n = 4, P = 0.001; Independent sample student t test). (J) Grik2b and Grik2c expression in CNS 12 h after abdomen injection of dsRNA targeting Grik2b or Grik2c (Grik2b: n = 4, P < 0.001; Grik2c: n = 4, P = 0.002; Independent sample student t test). (K) Ovipositi [file pbio.3003609.s012.docx]

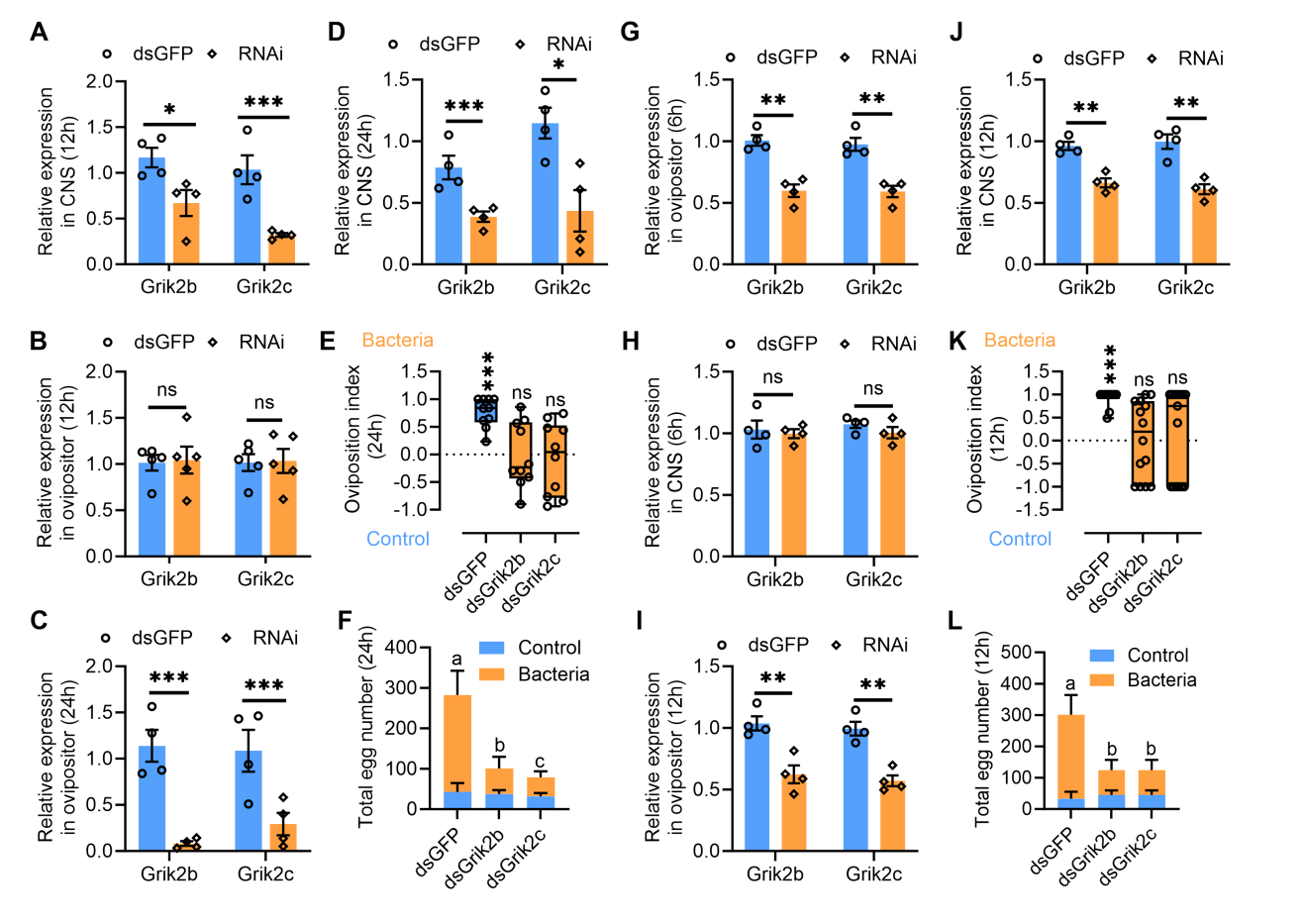


**S12 Fig. The effect of thorax or abdomen injection of dsRNA on the expression of KARs and oviposition.**

**(A)** Grik2b and Grik2c expression in CNS 12 hours after thorax injection of dsRNA targeting Grik2b or Grik2c (Grik2b: n = 4, *P* = 0.031; Grik2c: n = 4, *P* = 0.0042; Independent sample student’s *t* test).

**(B)** Grik2b and Grik2c expression in ovipositor 12 hours after thorax injection of dsRNA targeting Grik2b or Grik2c (Grik2b: n = 5, *P* = 0.8635; Grik2c: n = 5, *P* = 0.9123; Independent sample student’s *t* test).

**(C)** Grik2b and Grik2c expression in ovipositor 24 hours after thorax injection of dsRNA targeting Grik2b or Grik2c (Grik2b: n = 4, *P* < 0.001; Grik2c: n = 4, *P* = 0.0216; Independent sample student’s *t* test).

**(D)** Grik2b and Grik2c expression in CNS 24 hours after thorax injection of dsRNA targeting Grik2b or Grik2c (Grik2b: n = 4, *P* = 0.009; Grik2c: n = 4, *P* = 0.0144; Independent sample student’s *t* test).

**(E)** Oviposition preference to the gut bacteria strain added fruit of females with dsRNA of Grik2b and Grik2c injected into thorax for 24h (dsGFP: n = 10, *P* < 0.0001; dsGrik2b: n = 10, *P* = 0.9543; dsGrik2c: n = 10, *P* = 0.7336; Paired sample student’s *t* test).

**(F)** Total eggs laid by females with dsRNA of Grik2b and Grik2c injected into thorax for 24h (n = 10, *F*_(2,54)_ = 5.945, *P* = 0.0046, Two-way ANOVA).

**(G)** Grik2b and Grik2c expression in ovipositor 6 hours after abdomen injection of dsRNA targeting Grik2b or Grik2c (Grik2b: n = 4, *P* < 0.001; Grik2c: n = 4, *P* = 0.002; Independent sample student’s *t* test).

**(H)** Grik2b and Grik2c expression in CNS 6 hours after abdomen injection of dsRNA targeting Grik2b or Grik2c (Grik2b: n = 4, *P* = 0.6978; Grik2c: n = 4, *P* = 0.2639; Independent sample student’s *t* test).

**(I)** Grik2b and Grik2c expression in ovipositor 12 hours after abdomen injection of dsRNA targeting Grik2b or Grik2c (Grik2b: n = 4, *P* = 0.0041; Grik2c: n = 4, *P* = 0.001; Independent sample student’s *t* test).

**(J)** Grik2b and Grik2c expression in CNS 12 hours after abdomen injection of dsRNA targeting Grik2b or Grik2c (Grik2b: n = 4, *P* < 0.001; Grik2c: n = 4, *P* = 0.002; Independent sample student’s *t* test).

**(K)** Oviposition preference to the gut bacteria strain added fruit of females with dsRNA of Grik2b and Grik2c injected into abdomen for 12h (dsGFP: n = 10, *P* < 0.0001; dsGrik2b: n = 14, *P* = 0.8635; dsGrik2c: n = 15, *P* = 0.5822; Paired sample student’s *t* test).

**(L)** Total eggs laid by females with dsRNA of Grik2b and Grik2c injected into abdomen for 12h (n = 10 and 15, *F*_(2,70)_ = 5.973, *P* = 0.004, Two-way ANOVA).

The data underlying this figure can be found in S10 Data.
