# Supplementary material for: Grik2b and Grik2c kainate receptors regulate oviposition in Bactrocera dorsalis
Source: PLoS Biol. 2026 Feb 2;24(2):e3003609. doi: 10.1371/journal.pbio.3003609 (PMC12875582; doi:10.1371/journal.pbio.3003609)
Supplement: S1 Table — (DOCX) [file pbio.3003609.s013.docx]

**S1 Table. Prediction of hydrogen bond interactions between the glutamate receptors and the ligands.**

| Receptor | Ligand | Affinity （kcal/mol） | Dist from  rmsd l. b. | Best mode  rmsd u. b. |
| --- | --- | --- | --- | --- |
|  | Glutamate | -5.2 | 0.000 | 0.000 |
| Grik2b | D-AP5 | -13.5 | 0.000 | 0.000 |
|  | NBQX | -6.6 | 0.000 | 0.000 |
|  | Glutamate | -4.6 | 0.000 | 0.000 |
| Grik 2c | D-AP5 | -11.7 | 0.000 | 0.000 |
|  | NBQX | -7.2 | 0.000 | 0.000 |
