# Supplementary material for: Grik2b and Grik2c kainate receptors regulate oviposition in Bactrocera dorsalis
Source: PLoS Biol. 2026 Feb 2;24(2):e3003609. doi: 10.1371/journal.pbio.3003609 (PMC12875582; doi:10.1371/journal.pbio.3003609)
Supplement: S2 Table — (DOCX) [file pbio.3003609.s014.docx]

**S2 Table. Predication of binding site amino acid residue of glutamate receptors.**

| Receptor | Ligand | Biding sites | Common sites with glutamate |
| --- | --- | --- | --- |
| Grik2b | Glutamate | THR-57, GLY-58, THR-87, ARG-92 | NA |
|  | D-AP5 | ASP-85, THR-87, ARG-92, SER-137 | THR-87, ARG-92 |
|  | NBQX | ARG-56, THR-57, ARG-92, SER-137 | THR-57, ARG-92 |
| Grik2c | Glutamate | ARG-65, TYR-66, THR-95,  ARG-98, GLY-141 | NA |
|  | D-AP5 | TYR-66, THR-95, ARG-98, GLY-141 | TYR-66, THR-95, ARG-98, GLY-141 |
|  | NBQX | THR-95, ARG-98, GLY-141 | THR-95, ARG-98, GLY-141 |
