# Supplementary material for: Grik2b and Grik2c kainate receptors regulate oviposition in Bactrocera dorsalis
Source: PLoS Biol. 2026 Feb 2;24(2):e3003609. doi: 10.1371/journal.pbio.3003609 (PMC12875582; doi:10.1371/journal.pbio.3003609)
Supplement: S3 Table — (DOCX) [file pbio.3003609.s015.docx]

**S3 Table. Primer sequences used in this study.**

| Experiment | Primer name | | | Sequence (5’-3’) |
| --- | --- | --- | --- | --- |
| qPCR | | *RPL32*-F | CCCGTCATATGCTGCCAACT | |
|  |  | *RPL32*-R | GCGCGCTCAACAATTTCCTT | |
|  |  | *Grik2a*-F | ATATACGCGCTACTCTGCACT | |
|  |  | *Grik2a* -R | CGAACGCGATATTTCGGTTTCACT | |
|  |  | *Grik2b*-F | GTATGACTCCGGCTACAGGG | |
|  |  | *Grik2b*-R | CGTAAGCGCGTCTTCTTTCG | |
|  |  | *Grik2c*-F | AGGCAGACAGCGACATTGTT | |
|  |  | *Grik2c*-R | GCTCCCGGTTTGTCTCTTCA | |
|  |  | *Mhc-*F | TCCGAAATACGAAAAGGCCGA | |
|  |  | *Mhc-*R | AGGATTGATGGCAACGCAGA | |
|  |  | *Mlc-*F | CACTCCAGCTCCAGCTTCAA | |
|  |  | *Mlc -*R | CGGCAATCTGTTTCTGCGAG | |
|  |  | *Actin-2-*F | CGCCAACATTGTCATGTCCG | |
|  |  | *Actin-2-*R | TTTACGTTCTGGTGGGGCAA | |
|  |  | *Actin-3-*F | GGCGCCATCCACCATTAAGA | |
|  |  | *Actin-3-*R | CTGGGCCGGATTCATCGTAT | |
|  |  | *GluSN-*F | AAGCAACAACGTTAGACAGTG | |
|  |  | *GluSN*-R | CTGCCAAAACTCATAGCGCC | |
|  |  | *GluDH-*F | CCATGCGACATCTTTGTGCC | |
|  |  | *GluDH-*R | TTTATCGGCAGCTGGAGTGG | |
|  |  | *Vglut-*F | ACCGCTTATTTGGGCAGTTC | |
|  |  | *Vglut-*R | TCAATGTAACGTTGTTCCGAGC | |
| RNA Interference | | ds*Grik2a*-F | taatacgactcactatagggTATGGCGCTTTACTAGGCGG | |
|  |  | ds*Grik2a*-R | taatacgactcactatagggCCACCACCATGCATCTCCTT | |
|  |  | ds*Grik2b*-F | taatacgactcactatagggTCGCGATGAATACGAAATTGAGC | |
|  |  | ds*Grik2b*-R | taatacgactcactatagggTCTGTTGTTAAGGCGATCTTGA | |
|  |  | ds*Grik2b*-F2 | taatacgactcactatagggGATTCCGGTTACAGGGCGAA | |
|  |  | ds*Grik2b*-R2 | taatacgactcactatagggTGTGTGCGTTGACCGTTTTC | |
|  |  | ds*Grik2c*-F | taatacgactcactatagggTGAAGAGACAAACCGGGAGC | |
|  |  | ds*Grik2c*-R | taatacgactcactatagggAACTCCGGTGCACAATCCAA | |
|  |  | ds*Grik2b*-F2 | taatacgactcactatagggATCTGCTTGTGATCTCCGCC | |
|  |  | ds*Grik2b*-R2 | taatacgactcactatagggCGGTCTCGTTAGATTCGGCA | |
|  |  | ds*Mhc-*F | taatacgactcactatagggAACGCTAAGACCGTGCGTAA | |
|  |  | ds*Mhc-*R | taatacgactcactatagggTCTCTTGAGCGGTGAAACCC | |
|  |  | ds*Mlc-*F | taatacgactcactatagggAATCGCCGCCGATAAGGAAT | |
|  |  | ds*Mlc -*R | taatacgactcactatagggTTGCCGGTGAGCATCTCAAT | |
|  |  | ds*Actin-2-*F | taatacgactcactatagggACGTCGGTGATGAAGCTCAA | |
|  |  | ds*Actin-2-*R | taatacgactcactatagggGCCAAGTCCAAACGGAGGAT | |
|  |  | ds*Actin-3-*F | taatacgactcactatagggTGTGTGACGACGAAGTTGCT | |
|  |  | ds*Actin-3-*R | taatacgactcactatagggGGTGAGCAATACTGGGTGCT | |
|  |  | ds*GluSN*-F | taatacgactcactatagggTTCATTACCGATCGAAAGCC | |
|  |  | ds*GluSN*-R | taatacgactcactatagggGCATTCGGATTTGAGCATTT | |
|  |  | ds*GluDH*-F | taatacgactcactatagggAGCGAAAACGCTCACAAAAT | |
|  |  | ds*GluDH*-R | taatacgactcactatagggCCGGAGTGTACGATGTCCTT | |
|  |  | ds*GFP*-F | taatacgactcactatagggACTACCTGTTCCATGGCCAAC | |
|  |  | ds*GFP*-R | taatacgactcactatagggACTACCTGTTCCATGGCCAAC | |
|  |  | ds*Vglut*-F | taatacgactcactatagggCTCTTCGCTTTAGCCTTCTC | |
|  |  | ds*Vglut*-R | taatacgactcactatagggAAGCACGTTTCGGTGAGTTC | |
| FISH | | *Grik2b* | CTCATCGACTAACTTTAATCTCACAGAAGTTATATTCGCCCTGTAACCGG-Cy3 | |
|  |  | *Grik2c* | GACTGCGTAACCCTCATACCGTTCATTACCAGTGACATTTTGCTCCACCA-Cy3 | |
|  |  | *Vglut* | GAATAGACCCCAAATGCCGCCCCACTGTGCGATCACATTCATCCAGACAGC-5'FAM | |
| Proteolysis assays | | *Grik2b-*F | ACTAGTCGTAAAAAGTTTATAGTGACAACACGCTTCG | |
|  |  | *Grik2b-*R | GCATGCACTAGGACATTCAGGTTTAACAGTCGG | |
|  |  | *Grik2c-*F | ACTAGTACGAAGAAGTTGGTTTATCGTGTGGC | |
|  |  | *Grik2c-*R | GCATGCATTGTCCTCGCAATCCGTATCGT | |
| Electrophysiological experiments | | *Grik2b*-F | GGCAGATCTGATATCACTAGTATGCGTTTTAGCAACATTTTCG | |
|  |  | *Grik2b*-R | TGAGGCTGGTTTAGTGGTAACCTTAGTCATAGTAACGGCGACTGAGTA | |
|  |  | *Grik2c*-F | GGCAGATCTGATATCACTAGTATGAATATAAATACGAAATATTGGATTATTG | |
|  |  | *Grik2c*-R | TGAGGCTGGTTTAGTGGTAACCCTACATTTCATCTACTTTAATGATAGTGCC | |

Note: The lowercase letters represent the T7 promoter; The red sequence is the restriction site; The blue sequence is the homologous sequence of the plasmid.
