# Supplementary material for: Grik2b and Grik2c kainate receptors regulate oviposition in Bactrocera dorsalis
Source: PLoS Biol. 2026 Feb 2;24(2):e3003609. doi: 10.1371/journal.pbio.3003609 (PMC12875582; doi:10.1371/journal.pbio.3003609)
Supplement: Attachment — Submitted filename: Response to reviewers.docx [file pbio.3003609.s029.docx]

As you will see, the reviewers find the conclusions potentially interesting, however they also raise several concerns that would need to be addressed before we can consider the manuscript for publication. Reviewer 1 thinks that the data as presented doesn’t support the main conclusions and that the RNAi experiments should be strengthened, along with additional controls, better quantification of the experiments and behavioural data for Grik2a. Reviewer 2 asks for further insights into the neuronal mechanisms that controls egg-laying behaviour, an analysis of the function of the Grik2b receptor and increasing the sample size for the behavioural experiments among other points. Reviewer 3 mentions several points that would need to be clarified.

**Authors’ Response:**

Thank you for giving us the opportunity to revise our manuscript and for the constructive feedback from the reviewers. We sincerely appreciate the reviewers’ positive assessment of the potential interest of our work and their insightful comments and suggestions.

We have carefully considered all the points raised and have performed extensive additional experiments and analyses to address them. These revisions have significantly strengthened the manuscript, we believe. Below, you will find our point-by-point responses to each comment, detailing the changes made.

We hope that these revisions and clarifications now meet the standards for publication in PLoS Biology.

In addition, the Academic Editor has raised several points that would been to be also addressed. These include whether or not the knockdown of the receptors affect the overall vitality of the flies, as the observed phenotype (lowered oviposition, etc) could be simply an indirect consequence of that. The Academic Editor is also concerned about off-targets’ knockdown causing the phenotype.

**Authors’ Response：**

We thank the Academic Editor for raising these important points regarding the specificity of the phenotype and the overall health of the flies. We have addressed these concerns as detailed below.

To address the concern that the observed phenotypes (e.g., reduced oviposition) were an indirect consequence of reduced overall vitality; we assessed the general health and locomotion of the receptor-knockdown flies. We performed a negative geotaxis assay and found no significant difference in climbing ability between the knockdown and control groups. These data indicate that the knockdown flies are otherwise healthy and active. Therefore, the specific reduction in oviposition is unlikely to be a secondary consequence of general sickness or debilitation. See Figure S8, Line 97.

Regarding the concern of off-target effects, we have taken a standard genetic approach to confirm the specificity of our findings. We used two independent RNAi lines targeting non-overlapping regions of the receptor mRNA. Both lines recapitulated the same oviposition phenotype, making it highly unlikely that the effect is due to a shared off-target. This data is now included in the revised manuscript as Figure S7F-7I. Line 419-422.

**Reviewers' comments**

**Reviewer #1:**

The study proposes that two kainate type iGluRs (Grik2b, Grik2c) act in both glutamatergic CNS neurons and ovipositor neuromuscular junctions (NMJs) to regulate oviposition preference and egg number in Bactrocera dorsalis. Evidence cited includes ovipositor enriched transcriptomics, FISH and histology at ovipositor NMJs, FISH colocalization with Vglut in the CNS, RNAi knockdowns affecting behavior, docking plus MST/SPR ligand binding, limited Xenopus oocyte recordings, and small molecule antagonist injections. While the hypothesis is not uninteresting, the data as presented do not yet support the central conclusions.

**Authors’ Response：**

We sincerely thank you for the in-depth review of our manuscript and your highly constructive critical comments. Your rigorous standards regarding the relationship between the strength of evidence and the conclusions drawn are crucial for us to further refine this work and enhance its scientific rigor. We fully agree that establishing definitive mechanisms for such complex physiological functions across scales is a demanding task.

After carefully considering your comments, we have re-examined our data and the presentation of our conclusions. We believe that, although individual experiments may have limitations, the multi-faceted evidence we provide collectively forms a compelling chain of evidence supporting the core hypothesis that glutamatergic signaling regulates egg-laying behavior at multiple levels through Grik2b/2c. Here is our integrated argument:

**Genetic and Behavioral Causal Link:** CNS and ovipositor-specific knockdown of Grik2b/2c was sufficient to recapitulate the deficits in egg-laying preference and number, establishing its necessity in regulating this behavior within the central nervous system. This aligns perfectly with the evidence of Grik colocalization with Vglut in the CNS. See Figure 4.

**Pharmacological Congruence:** Application of Grik receptor antagonists at the whole-animal level reproduced the behavioral phenotypes observed in genetic knockdowns. This indicates that even in genetically unmodified wild-type flies, pharmacological blockade of this receptor can produce the same effects, significantly reducing the likelihood that the phenotypes result from off-target RNAi effects. See Figure 5K and 5L.

**Anatomical and Molecular Basis for Peripheral Action:** We detected Grik2b/2c transcripts at the ovipositor NMJ, providing the possibility for their direct role in regulating motor output peripherally. Ligand-binding experiments (MST/SPR) and functional data from oocytes demonstrate that these receptors can indeed form channels responsive to glutamate. See Figure 4.

**Synergistic Effect of the Evidence:** Most critically, these lines of evidence are mutually supportive and synergistic. The NMJ localization alone cannot prove functional importance, but when combined with the behavioral pharmacology and CNS evidence, a unified picture emerges: glutamate acts both as a key neurotransmitter in the CNS and as a muscle excitatory agent at the NMJ, with Grik2b/2c being key receptor molecules coordinating this process.

We acknowledge that direct electrophysiological evidence of NMJ function and more detailed biophysical characterization in the oocyte system are aspects of the evidence chain that can be further strengthened. Following your suggestion, we have explicitly acknowledged this limitation in the Discussion section and outlined a clear path for future research utilizing in vitro electrophysiological recordings and cell-type-specific rescue experiments to ultimately confirm this model. See line 266-272.

Concurrently, we have carefully revised the conclusive statements throughout the manuscript to ensure greater precision, for instance, changing phrases like "...proves that..." to "...provides strong supporting evidence that...", to ensure our claims precisely match the demonstrative strength of the current data. See line 19-21, line 219-221.

We believe that through these revisions and clarifications, the scientific rigor of the manuscript have been significantly enhanced, allowing its importance and limitations to be presented more clearly to the reader. We thank you again for helping us strengthen this work.

**Major issues**

1. The inference that Grik2b/c are required relies heavily on RNAi, with limited assessment of off target effects. Stronger evidence would include (i) multiple, non overlapping RNAi constructs with concordant phenotypes, (ii) rescue with RNAi resistant cDNA, and/or (iii) CRISPR loss of function plus genetic rescue. Clarify pharmacology: the antagonist used appears inconsistent with kainate receptor selectivity; specify its known target profile and justify its use here.

**Authors’ Response：**

Thank you very much for your suggestion. In fact, we have also taken into account the potential off-target effects in RNAi. To avoid off-target effect in RNAi, we have performed off-target prediction of RNAi target sites by setting the threshold of off-target bases to 19 bp (Kulkarni et al., 2006, Nature methods). See line 419-422.

Since CRISPR loss of function of Grik2b/c has lethal effect on the flies (The female insects lay very few eggs, making it difficult to obtain mutant homozygous strains), non-overlapping RNAi constructs were done as suggestion. As predicted, the concordant phenotypes were observed. See Figure S7F-7I, line 419-422.

As for rescue with RNAi resistant cDNA, we fully agree with your perspective that the functional rescue experiment serves as the most robust evidence for establishing a causal relationship in gene function. We have also thoroughly explored the feasibility of conducting such rescue experiments in *B. dorsalis*. However, as a non-model organism, this species presents several technical challenges that are currently difficult to overcome. At present, a stable rescue gene expression system has not been established for this species, and the necessary tools for genetic-level rescue experiments are lacking. We have explicitly acknowledged this limitation in the Discussion section. See line 271-276. Although functional rescue experiments could not be performed, we have implemented multiple rigorous alternative strategies in our study to maximize the validation of RNAi specificity and ensure the reliability of our conclusions. We additionally synthesized independent dsRNAs targeting distinct sites of the gene as your suggestion. As shown in Figure S7F-7I, these independent dsRNAs effectively knocked down the target gene and consistently induced reproducible phenotypes.

In summary, while the absence of functional rescue experiments is admittedly a limitation, we believe that our multi-sequence, multi-level validation approach has provided compelling evidence for the role of Grik2b/c in egg-laying. We earnestly request that you consider both the objective technical challenges we faced and the substantial data we have provided in support of our current conclusions. Once again, we thank you for your valuable feedback, which has helped improve the quality of our work.

For clarifying pharmacology, in studies on vertebrates, D-AP5 and NBQX are selective antagonists for NMDA receptors and AMPA/Kainate receptors, respectively (Watkins and Evans, 1981; Randle et al., 1992). Research in *Drosophila*, however, has shown that kainate receptors can be inhibited by D-AP5. Phylogenetic analysis reveals that Grik2b and Grik2c share high similarity with kainate receptors in *Drosophila* (Li et al., 2016). Therefore, we selected these two antagonists to assess their inhibitory effects on Grik2b/c. See line 193-199.

Thanks again for your comments and suggestions. We hope the revision and explanation will meet with approval.

2. The attribution of reduced egg laying to ovipositor iGluR is correlative. Survey additional candidate loci (e.g., oviduct musculature) and, where feasible, apply tissue restricted manipulations to localize function.

**Authors’ Response：**

We thank the reviewer for this critical and constructive comment. We fully agree that establishing a causal link beyond the current correlative data is essential. We appreciate the suggestion to explore additional candidate loci and to perform tissue-specific functional analyses. In response, we have additional experiments to strengthen our conclusions:

We extended our study to rigorously examine other factors. Specifically, we noted a marked high expression of genes associated with muscle development and contraction in the female ovipositor. Knockdown of these genes also resulted in reduced egg laying. However, interference with Grik2b/c did not alter the expression of these muscle development- and contraction-related genes, suggesting that the effect of Grik2b/c on oviposition is independent of the transcriptional regulation of these muscle-associated genes. See Figure S9.

We acknowledge that tissue-restricted manipulation is the gold standard for pinpointing the site of gene action. However, generating the requisite tissue-specific knockout/knockdown tools (e.g., a conditional allele or a highly specific Cre driver line) in our model system is technically challenging and would require a timeframe beyond the scope of this revision. To address the reviewer's excellent point about localization of function as rigorously as possible, we added a dedicated section in the Discussion to explicitly address the limitation of correlation and carefully interpret our findings. See line 271-276.

Furthermore, we conducted additional experiments to examine receptor expression in the ovipositor and central nervous system (CNS) shortly after abdominal injection. The results revealed that injecting dsRNA into the abdominal region near the ovipositor specifically reduced receptor expression in the ovipositor within 6 hours, without affecting receptor levels in the CNS. Under these conditions, we found that knocking down receptor expression did not influence oviposition preference but significantly decreased the egg-laying quantity. These findings further demonstrate the role of receptors expressed in the ovipositor in regulating egg-laying output. See Figure 4O and 4P, line 182-186.

We believe that these additional experiments and nuanced discussion will significantly strengthen our manuscript by providing a much deeper mechanistic insight and directly addressing the correlative nature of our initial claim. The new data will allow us to present a more compelling model for how the iGluR regulates egg-laying.

3. The oviposition phenotype is described at a very coarse level. High magnification, quantitative kinematics (e.g., ovipositor extrusion frequency, duration, bout structure) would clarify if and how iGluR disruption alters the motor program.

**Authors’ Response：**

We thank the reviewer for this excellent suggestion. We agree that a quantitative, kinematical analysis of the oviposition motor program will provide much deeper insight into the specific nature of the defect caused by iGluR disruption. In direct response to this comment, we have performed the following experiments:

**High-Resolution Video Capture:** We set up a high-speed/high-magnification video recording system to capture detailed ovipositor movements in freely behaving animals. This allows us to resolve fine-scale motor patterns with high temporal precision. See Video 1 and 2, line 97-99.

**Quantitative Kinematic Analysis:** We analyzed the recorded videos. Specifically, we measured the following parameters in both control and experimental genotypes. Ovipositor Extrusion Frequency: The number of extrusion events per unit time. Duration: The duration of individual extrusion bouts. See Figure 2C and 2D, line 97-99.

We are confident that this detailed analysis allows us to move beyond the coarse description of "reduced egg-laying" and precisely determine how the motor program is altered.

We have incorporated these new quantitative data into the Results section of the revised manuscript. This would significantly strengthen our findings by providing a mechanistic explanation for the behavioral phenotype.

**Minor / specific issues**

1.Behavioral data for Grik2a are missing. If "redundant roles" are claimed, double knockdown (Grik2b+Grik2c) should exhibit a stronger than either single (often super additive) phenotype, state and test that prediction explicitly.

**Authors’ Response：**

Since our previous data showed that Grik2a is highly expressed only in the female thorax, a pattern distinct from the specific ovipositor expression of Grik2b/c (See Figure 1G-1I), we hypothesized that Grik2a is not involved in regulating oviposition. Therefore, it was not subjected to further investigation initially. In response to your concern, we have now included additional behavioral data for Grik2a in the revised manuscript. The results confirm that Grik2a has no impact on egg-laying. See Figure S7J-7L, line 105-106.

We are grateful for the opportunity to correct our description of the Grik2 receptor function. Our intended meaning was that these receptors possess the dual function of modulating both the quantity of eggs laid and the egg-laying preference. We have rephrased the relevant results in the revised manuscript accordingly and thank you for bringing this to our attention. See the revised title. Line 221-223.

We hope the revision and explanation will meet with approval.

2. In Fig. 2n (and 2j), both control and experimental groups lay far fewer eggs than in Fig. 2i. Such baseline drops can bias preference indices; report raw counts, normalize within day/batch, and analyze with models that account for baseline variation.

**Authors’ Response：**

We thank the reviewer for this critical insight regarding the potential confounding effect of baseline egg-laying variation on our preference indices. We agree that this is an important issue. In response to your query regarding egg-laying variation, we wish to clarify that it stems from intrinsic seasonal sensitivity in this tropical insect, *Bactrocera dorsalis*. Although maintained in a constant environment, its physiology and behavior still fluctuate. The original Figure 2i experiment was performed in early summer, when fecundity is naturally highest, explaining the discrepancy with experiments later in the year. To resolve this conclusively, we have now repeated the Figure 2i experiment to provide robust data. See Figure 2G. We hope the revision and explanation will meet with approval.

3. Include sense probe (and ideally RNase) controls and specify the exact ovipositor subregions sampled; add a schematic to anchor tissue locations.

**Authors’ Response：**

We thank the reviewer for these critical suggestions to improve the rigor and clarity of our in situ hybridization data. We agree that these additions are essential for validating the specificity of our signal and for providing precise anatomical context. We have addressed these points as follows:

**Inclusion of Sense Probe and RNase Controls:**

We apologize for this omission. To definitively demonstrate the specificity of our antisense probe signal, we performed and include the following control experiments in the revised manuscript. Sense Probe Control: We hybridized parallel tissue sections with sense probes for the Grik transcripts. We did see only background-level or absent staining with this control, which confirmed that the signal from our antisense probe is specific to the mRNA of interest. See Figure S5E. RNase Treatment Control: We treated an additional set of tissue sections with RNase A prior to hybridization with the antisense probe. The elimination of staining in these RNase-treated sections provided conclusive evidence that the detected signal originates from RNA. See Figure S5F.

**Specification of Ovipositor Subregions and Addition of a Schematic:**

We agree that the description of the sampled tissue was insufficiently precise. Detailed Anatomical Description: We revised the Methods and Results sections to explicitly state the exact subregions of the ovipositor from which the samples were derived. This included reference to specific anatomical landmarks. Anatomical Schematic: We have added a clear schematic diagram illustrating the ovipositor anatomy and explicitly indicating the specific subregions that were sampled for our analysis. See Figure 2B. This will provide readers with an unambiguous spatial framework for interpreting our results.

We are confident that these additions will significantly strengthen the validity of our in situ hybridization findings and greatly enhance the clarity and reproducibility of our work.

4. Fig. 4k implies near abolition of VNC activity after knockdown, but the format is not acceptable for inference. Provide n per group, ROI definitions, ΔF/F₀ quantification, motion correction, and appropriate statistics across biological replicates.

**Authors’ Response：**

We thank the reviewer for this critical comment regarding the presentation and quantification of our calcium imaging data in Fig. 4k. We agree that the current representative trace format, while suggestive, is insufficient for rigorous statistical inference. We have now performed a comprehensive re-analysis of the full dataset and will revise the figure and text accordingly to include the following essential information and controls:

**Sample Size (n) and Biological Replicates:** We have explicitly stated the number of recorded ROIs per group in the figure legend. All statistical analyses were performed across biological replicates. See Figure 4K and 4L.

**ROI Definition and Selection Criteria:** We have detailed in the Methods section how Regions of Interest (ROIs) were defined. Specifically, ROIs were manually drawn to correspond to the somata of individual neurons within the target region of the VNC. See Figure 4K. Line 406-416.

**Quantification of ΔF/F₀:** The fluorescence intensity (F) for each ROI was calculated by ImageJ 1.54d. See line 411-416.

**Appropriate Statistical Analysis Across Replicates:** We have added a quantified summary plot. The new Figure 4L showed the mean ± SEM of the peak ΔF/F₀ response for both control and knockdown groups.

We believe these substantial revisions, which move from a representative trace to a fully quantified and statistically robust analysis, will firmly support our conclusion that iGluR knockdown profoundly reduces VNC activity. We thank the reviewer for pushing us to elevate the standard of our evidence.

**Reviewer #2:**

This paper identifies the neurotransmitter glutamate as playing a role in oviposition behavior of the oriental fly Bactrocera dorsalis. Studying oviposition behavior of this species is of interest since it is an agricultural pest affecting fruit production by laying its eggs in the fruits and very little is known on the neuronal mechanisms governing this behavior. The females use an elongated ovipositor to deposit their eggs deep inside the fruits and this organ appears to be also involved in sensing egg-laying substrates.

The authors investigate glutamate signaling after having identified by transcriptomic analysis 3 glutamate receptors (Grik2a, 2b and 2c) as being highly expressed in the ovipositors of gravid females. They focus on 2 of these receptors, Grik2b and 2c, and demonstrate via several biochemical experiments that they indeed bind to glutamate and are able to induce currents when expressed in Xenopus oocytes. They also document expression patterns of the receptors in the Central Nervous System and ovipositor by in situ hybridization.

They then demonstrate via RNAi knockdowns that Grik2b and 2c regulate oviposition behavior. They confirm these results by manipulating glutamate levels (GluDH, GluSN and vGlut RNAi knockdown) and by injecting receptor inhibitors into the flies (D-AP5, NBQX) in the same behavioral assays. Notably, the authors argue that they can distinguish two separate functions on oviposition behavior by glutamate signaling: Grik receptors expressed in the CNS appear to participate in determining egg-laying substrate preference (favoring substrates containing bacteria), while Grik receptors in the ovipositor probably directly control muscle contractions to expel the eggs since they are expressed in muscle cells (i.e.: Neuro Muscular Junction).

Overall, the data is well presented with appropriate controls. However, while this paper uses a challenging model organism and manages to perform functional manipulations (RNAi knockdowns) to demonstrate the involvement of glutamate signaling, it provides relatively little insight into the neuronal mechanisms controlling this behavior. In fact, glutamate has already been implicated in driving the egg-laying motor program in Drosophila (e.g. Cury and Axel 2023, Oliveira-Ferreira et al. 2023) but this is not mentioned in this paper. The paper seems to allude to potential applications for pest control via the use of Grik receptor inhibitors but does not explore this idea further.

**Authors’ Response：**

We sincerely thank you for reviewing our manuscript and providing valuable feedback. Your encouragement and insightful comments have been crucial for further refining this work. We have carefully revised the manuscript based on your suggestions.

We fully agree with the reviewer's opinion that delving into the neural mechanisms of behavior is essential. To address this point, we have undertaken the following work in the revised manuscript:

**We conducted a more in-depth behavioral analysis.** Beyond the original egg-laying counts, we have now quantified the kinetic parameters of oviposition movements. These new data analyses reveal that knockdown of Grik signaling not only affected the quantity of eggs laid but, more specifically, impaired the initiation of the oviposition motor program. This provides deeper mechanistic insight into its precise role in motor pattern generation. See Figure 2C and 2D, Video 1 and 2, Line 97-99.

**We have expanded the Discussion section.** We specifically discuss potential specific neural nodes where Grik signaling might act and propose future experimental directions, such as cell-type-specific rescue experiments or calcium imaging, to directly test these hypotheses. We believe these additions significantly strengthen the exploration of the underlying neural mechanisms. See line 264-271.

Regarding the omission of key references, we thank the reviewer for pointing out this oversight, and we apologize. We have carefully read and cited both important papers (Cury and Axel 2023, Oliveira-Ferreira et al. 2023). In the Introduction of the revised manuscript, we now cite them to establish the conserved role of glutamate in egg-laying. See line 37-39. More importantly, in the Discussion, we now explicitly compare our findings with these studies. We highlight that our research in *B. dorsalis* confirms the broad importance of glutamatergic signaling, specifically through Grik receptors. This strongly resonates with findings in *Drosophila*, suggesting this signaling pathway may be deeply evolutionarily conserved in insect egg-laying behavior. See line 223-227.

Concerning the mention of the potential application of Grik receptor inhibitors in pest control without further exploration, we agree that the previous statement was overly speculative. In the revised manuscript, we have made the following adjustments: We have modified the wording regarding application prospects throughout the text, particularly in the concluding part of the Discussion, shifting the tone from direct "application" to the more appropriate "implications for future pest control strategies." See line 277-282. Furthermore, we have added a dedicated paragraph in the Discussion on "Challenges and Prospects for Translational Application." Here, we not only discuss the potential of the Grik target as a novel insecticide target but also seriously address the potential obstacles it may face in practical application, making our discussion more balanced and rigorous.

Specific comments:

1. It is not clear to me why the authors didn't investigate the function of the Grik2a receptor.

**Authors’ Response：**

Since our previous data showed that Grik2a is highly expressed only in the female thorax, a pattern distinct from the specific ovipositor expression of Grik2b/c, we hypothesized that Grik2a is not involved in regulating oviposition. See Figure 1G-1I. Therefore, it was not subjected to further investigation initially. In response to your comment, we have now included additional behavioral data for Grik2a in the revised manuscript. The results confirm that Grik2a has no impact on egg-laying. See line 105-106, Figure S7J-7L.

2. The authors observe phenotypes when knocking down either one the Gri2b and 2c receptors throughout the paper and they mention "function redundancy of kainite receptors" in their ms title but they don't discuss the implications. How is this working? If both receptors are essential, they are not redundant…

**Authors’ Response：**

We are grateful for the opportunity to correct our description of the Grik2 receptor function. Our intended meaning was that these receptors possess the dual function of modulating both the quantity of eggs laid and the egg-laying preference. We have rephrased the relevant results in the revised manuscript accordingly and thank you for bringing this to our attention. See the revised title.

The authors should control for specificity of their dsRNA probes, showing that dsRNA against Gri2b do not affect Grik2c levels and vice versa.

**Authors’ Response：**

We thank the reviewer for this excellent suggestion. We agree that demonstrating the specificity of our dsRNA probes is essential to strengthen our conclusions. We apologize for this oversight in our initial submission. Following the reviewer's advice, we have now performed the requested control experiments. We designed and synthesized new batches of dsRNA targeting Gri2b and Grik2c, and treated separate sample sets to assess cross-reactivity using qRT-PCR. The new results, now presented in Figure S7A-7D of the revised manuscript, clearly demonstrate that: dsRNA against Gri2b effectively silences Gri2b expression, while Grik2c mRNA levels remain unchanged compared to the control group. Similarly, dsRNA against Grik2c specifically reduces Grik2c expression without altering Gri2b levels. These data robustly confirm the specificity of our knockdown approach and support our interpretation that the observed phenotypes are due to the specific loss of the targeted gene. We have updated the Methods and Results sections accordingly and believe this new figure significantly improves the manuscript.

3. The sample sizes for many behavioral experiments appear quite low (e.g.: n=5 replicates in Fig 2l-m, Fig 5k-l)

**Authors’ Response：**

We have increased the number of experimental repeats/replications in the revised manuscript. See Figure 2 H and Figure 5K (n = 15). Thank you for your suggestion.

4. Lines 217-218: I don't understand the authors' argument. The fact that Grik receptors respond to glutamate does not imply that they should be expressed in glutamate-producing neurons.

**Authors’ Response：**

We thank the reviewer for raising this important point. We acknowledge that our original statement was overly simplistic. Our hypothesis is not solely based on the ligand-receptor relationship but is also supported by prior evidence in similar neural systems. Several studies have documented the co-expression of Grik subunits with vesicular glutamate transporters (vGluT) in specific brain regions, suggesting a functional coupling in these circuits (Jane et al., 2009, Neuropharmacology; Huettner et al., 2003, Progress in Neurobiology). Therefore, our argument is grounded in this specific biological context rather than a general principle. We have revised the text to more precisely frame our hypothesis, citing this supporting literature and avoiding the broad implication that the reviewer rightly questioned. See line 155-160.

5. Fig 4a-d: the authors detect Grik2b/2c expression in the CNS but do not detect them by qPCR in the thorax in Fig 1h-I. Do they have an explanation?

**Authors’ Response：**

We thank the reviewer for this astute observation regarding the apparent discrepancy between our qPCR and FISH data for Grik2b/2c. This is not due to an inconsistency in the data, but rather reflects the fundamental differences in sensitivity and resolution between these two techniques when applied to complex tissues.

**qPCR on bulk tissue**: The qPCR analysis in Fig. 1H and 1I was performed on RNA extracted from the entire thorax, which is a heterogeneous mixture of tissues (e.g., muscle, cuticle, fat body, trachea, and a small number of neurons). If Grik2b/2c expression in the thorax is restricted to a sparse population of neurons, its signal in the total RNA pool would be extremely low, potentially falling below the robust detection limit of our qPCR assay. The signal is effectively "diluted" by the vast majority of non-expressing cells.

**Cellular resolution of FISH**: In contrast, the FISH in Figure 4 allows for the spatial visualization of transcript location (The signal for Grik2b/2c was indeed detected only in a subset of cells). It is exquisitely capable of detecting a signal within a small subset of positive cells, even if those cells constitute a tiny fraction of the total tissue volume. Therefore, the presence of Grik2b/2c signal in specific neurons within the CNS (and potentially in peripheral neurons, though not the focus of this figure) is entirely consistent with its undetectable level in bulk thoracic qPCR.

6. Fig 4e-f: it is difficult to understand what we are looking at. The authors argue that Gri2b/2c do not colocalize with vGlut but in fact there is a bright spot in the center of the image in both cases with strong green and red signal. Is this a neuron(s)?

**Authors’ Response：**

We thank the reviewer for their careful examination of these images and for raising this important point. The bright spot in the center of the image should not be a neuronal cell body, and we appreciate the opportunity to clarify our interpretation. In this bright spot region, we did not observe corresponding nuclear signals (DAPI staining signals). We speculate that this is likely autofluorescence, which may originate from the intrinsic chemical components of the biological sample, such as elastin and collagen fibers, or certain neurotransmitters and flavins. We hope our explanation will meet with approval.

7. Fig 4k: It is also difficult here to understand what we are looking at. Is this the whole VNC? Only part of it?

Isn't it surprising that knocking down signaling of one neurotransmitter has such profound effects on calcium levels? Also, the images shown are only a snapshot, what are the temporal dynamics?

**Authors’ Response：**

Thank you very much for your thoughtful and constructive comments on our manuscript. Your insights have helped us to improve the clarity and rigor of our work. We have addressed all points raised, and our point-by-point responses are detailed below. Changes in the manuscript are highlighted in red.

**Reviewer Comment 1: It is also difficult here to understand what we are looking at. Is this the whole VNC? Only part of it?**

We thank the reviewer for pointing out this lack of clarity. Fig 4k indeed shows a representative region of the ventral nerve cord (VNC), specifically the cephalothoracic ganglion. We apologize for the ambiguity. We have now clearly labeled the region and added a schematic diagram in the revised figure to provide better spatial context for the reader. See Figure 4K.

**Reviewer Comment 2: Isn't it surprising that knocking down signaling of one neurotransmitter has such profound effects on calcium levels?**

The reviewer raises an excellent point regarding the profound effects observed upon knocking down a single neurotransmitter's signaling. We agree that this is a striking and significant finding. We believe there are several, non-mutually exclusive explanations for this. **Central Role of glutamate:** glutamate is known to be a primary excitatory neurotransmitter in the insect VNC, modulating broad neuronal circuits (Burrows, 1996, Oxford University Press; Bicker et al., 1988, Journal of Neuroscience). Therefore, perturbing its signaling is expected to have widespread network-level consequences, which are reflected in the population calcium signals we measured. **Network Amplification:** The VNC operates as a highly interconnected network. A perturbation in one node, especially one involving a key neurotransmitter, can be amplified through recurrent connections, leading to large-scale changes in activity dynamics. Our data supports this view, as we see coordinated changes across multiple regions. **Tonic Signaling vs. Phasic Signaling:** The knockdown may be affecting not only fast synaptic transmission but also tonic, ambient levels of the neurotransmitter, which are crucial for setting the overall excitability of the network. This could explain the global shift in calcium baseline/response we observed.

**Reviewer Comment 3: Also, the images shown are only a snapshot, what are the temporal dynamics?**

Thank you for raising this very important question. We fully agree that understanding the spatiotemporal dynamics of calcium signals is crucial for a comprehensive understanding of neural circuit function. However, significant technical challenges currently prevent us from achieving high spatiotemporal resolution in vivo calcium imaging in the non-model insect species used in this study. These are primarily due to: **Lack of Available Genetic Tools:** Unlike model organisms such as Drosophila melanogaster, there are currently no mature, neuron-type-specific genetic driver systems (e.g., analogous to the Gal4/UAS system) or other stable genetic driver lines available for the B. dorsalis. This prevents cell-type-specific expression of GCaMP. Our current imaging method is conducted at the population neuronal level. **Limitations in Optical Access and Signal-to-Noise Ratio:** The anatomical structure of the B. dorsalis nervous system and its opaque exoskeleton make long-term, high-frame-rate imaging in behaving, freely moving individuals exceptionally difficult. The signal-to-noise ratio of our current method is insufficient to reliably resolve temporal dynamics more refined than the "overall activity increase during stimulation" (e.g., individual peaks or oscillations). Given these technical constraints, the primary goal of our study was to address a more fundamental yet critical question: to determine whether a specific neuromodulatory signal has a global impact on the overall excitability level of the VNC during key behavioral states. Although these are "snapshot" measurements, the quantitative comparison of peak amplitude and overall active regions we provide offers clear and compelling evidence for this central question. Our data show that knocking down glutamatergic signaling indeed causes a significant and consistent alteration in network-level activity, which is itself an important finding.

We thank the reviewer again for this insightful comment. Resolving the temporal dynamics is undoubtedly a critical next step for our field. Based on this, we plan the following future research:

We will attempt to establish genetic manipulation tools in the *B. dorsalis* using methods such as novel viral vector approaches. Once such tools are established, we will immediately proceed with high-speed, cell-resolution functional imaging to precisely parse the millisecond-level dynamics of glutamatergic signaling in processes such as motor pattern generation and sensory integration.

We have incorporated this perspective and the related technical challenges as an important part of our future work in the concluding section of the Discussion in the revised manuscript See line 264-271, hoping to inspire fellow researchers.

Once again, we thank you for prompting us to consider the limitations and future directions of our work more deeply.

8. Fig 4o: the authors argue that CNS-specific knockdown of Grik2b/2c (12h after thoracic dsRNA injection) does not affect the egg-laying rate but in fact the rate is quite low, even in controls (about 100 eggs on average whereas it is usually around 200 in other experiments). Can they provide an explanation? Is it related to the shorter time of recovery after dsRNA injection?

**Authors’ Response：**

Thank you for pointing out this important observation regarding the egg-laying data in Figure 4o. We agree that the egg-laying rate in the control group of this experiment (approximately 100 eggs) is indeed lower compared to the average of about 200 eggs observed under other conditions in our laboratory.

We believe this difference is primarily related to the specific operational procedures unique to this experiment. To ensure behavioral detection at the specific time point of 12 hours after dsRNA injection, the recovery period for the flies after thoracic injection was relatively short. We speculate that the physiological stress from the injection procedure itself, combined with the shorter recovery period, are likely the main causes for the temporary and systematic reduction in egg-laying rate observed across all experimental groups, including the controls. Such transient behavioral suppression caused by procedural stress has been documented in insect ethological studies.

Furthermore, we cannot rule out that minor batch-to-batch variations or environmental factors may have also contributed to some extent. As mentioned in our response to a previous reviewer's question, *B. dorsalis* can exhibit significant seasonal variations in egg-laying capacity, and since our experiments were conducted over an extended period, seasonal factors could potentially have influenced the egg-laying rates.

However, we believe this systematic shift does not affect the core conclusion drawn from this experiment. The aim of this experiment was to assess the relative impact of CNS-specific knockdown of Grik2b/2c on the egg-laying rate. The key point is that both the control and knockdown groups were handled under identical conditions (same injection procedure, same recovery time, parallel behavioral testing). Therefore, the absence of a significant difference in egg-laying rate observed between the two groups strongly indicates that, within this specific experimental timeframe, the knockdown of Grik2b/2c did not exert an additional, specific effect on egg-laying behavior.

Following your suggestion, we will add a note in the corresponding figure legend or results section of the manuscript explicitly stating that the baseline egg-laying rate in this experiment may have been influenced by the injection procedure and the short recovery window, thereby alerting readers to this context. We thank you again for this valuable comment, which has enhanced the clarity and rigor of our data presentation.

9. Fig 5k-l: the authors inject the Grik inhibitors in the flies but this is not applicable in the field. Have they tried feeding the flies with these inhibitors?

**Authors’ Response：**

Thank you for raising this highly valuable perspective. We completely agree with your observation that the method of microinjection for drug delivery is indeed impractical for the field control of *B. dorsalis*.

In this study, our primary scientific objective was to achieve "proof-of-principle"-that is, to definitively answer the question: Does inhibiting glutamate receptor Grik activity in vivo suffice to cause measurable deficits in the egg-laying behavior of the flies? To test this scientific hypothesis most directly and reliably, we selected the thoracic injection route for drug administration. This approach ensures the accurate delivery of the inhibitor at a known dosage and maximizes its chance of reaching the central nervous system, thereby circumventing the complex variables introduced by uncertainties such as oral absorption efficiency, metabolic degradation, or blood-brain barrier permeability. For this foundational stage of mechanistic investigation, this methodological choice is rational and necessary.

Regarding the feeding method you suggested, we have not yet attempted to administer the drug via feeding. This is primarily because, as mentioned previously, the core focus of this research phase is mechanistic exploration. We recognize that developing an effective oral delivery strategy is a significant technical challenge in itself, involving complex pharmacokinetic studies that fall outside the current scope of this manuscript. However, your suggestion points us towards a clear and important direction for future research.

We firmly believe that fundamental scientific discoveries form the cornerstone of applied technological development. The significance of our study lies in its first demonstration that the Grik receptor is a valid molecular target for modulating egg-laying behavior in the B. dorsalis. This discovery provides crucial target information and a theoretical basis for future development of more practical pest control strategies. Subsequent research can build upon our findings to screen or design Grik receptor antagonists with better oral absorption and stability, or to explore novel technologies such as nanocarriers to facilitate their delivery in the field.

Following your suggestion, we will add a section to the Discussion of the revised manuscript explicitly stating the limitations of the current injection method for field application and outlining the potential and challenges of developing future delivery strategies based on feeding or spraying. See line 277-282.

Once again, we thank you for prompting us to extend our research perspective from laboratory mechanisms towards field application.

**Reviewer #3:**

In the current study, the authors investigated the roles of two kainate receptors (Grik2b and Grik2c) in regulating oviposition in Bactrocera dorsalis, an agricultural insect pest, proposing that glutamatergic neurons mediate oviposition preference while ovipositor NMJs control egg-laying quantity. The authors employed multiple approaches (transcriptomics, FISH, time control RNAi, LBD, electrophysiology, behavioral assays, and pharmacological inhibition). I found this interesting manuscript to be well-written and described. The findings in this study are valuable for helping us better understand the neural conduction mechanism of the insect oviposition behaviors and have practical implications for pest control strategies. The results should be useful to many in the field.

I have a couple of extra questions about the work,

1. The author should explain whether the additional independent non-overlapping dsRNA for targets is necessary to exclude the off-target RNAi effects.

**Authors’ Response：**

We thank the reviewer for raising this critical point regarding the necessity of controlling for off-target effects in RNAi experiments. We agree that this is an essential validation step. In the revised manuscript, we designed and tested two independent, non-overlapping dsRNAs for the target gene Grik2b/c. As shown in Figure S7F-7I, both dsRNAs elicited the same phenotype, whereas the control dsRNA did not. The consistency of the phenotype across two distinct dsRNAs that target different regions of the transcript makes it highly unlikely that the observed effects are due to off-target RNAi effects, thereby strongly supporting the conclusion that the phenotype is specific to the knockdown of Grik2b/c.

2. Even though the current study is focused on oviposition preference and egg-laying quantity, have the authors confirmed the retained eggs within the reproductive tract (such as ovaries) before/after injection to identify any effects on egg production?

**Authors’ Response：**

We fully agree with the reviewer's point. We dissected the ovaries of the injected females and counted the number of eggs in the ovaries. The results showed that the injection did not affect the number of eggs in the ovaries, thereby supporting our conclusion that the observed effects are related to central regulation rather than reproductive capacity. See Figure S7E.
